# Bioavailability of Bioactive Compounds from Reconstituted Grapefruit Juice as Affected by the Obtention Process

**DOI:** 10.3390/molecules28072904

**Published:** 2023-03-23

**Authors:** María del Mar Camacho, Juan José Martínez-Lahuerta, Eva García-Martínez, Marta Igual, Nuria Martínez-Navarrete

**Affiliations:** 1 Food Investigation and Innovation Group, Food Technology Department, Universitat Politècnica de València, Camino de Vera s/n, 46022 Valencia, Spain; 2 CA Juan Llorens, Departamento Valencia-Hospital General, Consellería de Sanitat Universal i Salud Pública, Generalitat Valenciana, 46008 Valencia, Spain; 3 I-Food IAD, Universitat Politècnica de València, Camino de Vera s/n, 46022 Valencia, Spain

**Keywords:** vitamin C, naringin, narirutin, naringenin, antioxidant capacity, grapefruit powder, freeze-drying, spray-drying, blood serum

## Abstract

Much attention has been paid to the health benefits of including fruits and vegetables in the diet. However, for the compounds responsible for this beneficial effect to be effective at the level of the human organism, they must be available for absorption after digestion. In this sense, in vivo studies are needed to demonstrate the bioavailability of these compounds and their physiological activity. In order to provide information in this regard, this study collects data on the levels of vitamin C (VC) and naringenin (NAG) in the blood serum of the 11 volunteer participants in this trial, before and after consuming two different grapefruit juices. The juices were prepared by rehydrating the grapefruit powder obtained by freeze-drying (FD) the fruit puree or by spray-drying (SD) the liquefied grapefruit. No significant differences (*p* > 0.05) neither by juice nor by participant were observed in any case. The mean relative increase of VC, NAG and the radical scavenging ability (RSA) in blood serum due to grapefruit juices intake was 12%, 28% and 26%, respectively. Just VC showed a positive and significant Pearson’s correlation with RSA. The mean bioavailability of VC was quantified as 1.529 ± 0.002 mg VC/L serum per 100 mg of VC ingested.

## 1. Introduction

Consumer attention is currently focused on healthy eating habits. Fruit juices can be clearly included in a healthy diet as they contain different health-promoting bioactive compounds, mainly VC and phenolics, which contribute to antioxidant capacity. Nevertheless, the consumer also looks for high-quality, stable and easy to handle foods. In this sense, fruit powder obtained from its edible part may be an interesting alternative to be consumed, for example, as juice after rehydration. Different techniques can be used to obtain powdered foods, with FD and SD providing the highest quality products. A lot of studies could be cited that have been carried out to evaluate the impact of different food processes on product quality. Nevertheless, the studied quality attributes are related to the composition, physical, chemical or biochemical properties or sensory aspects, among others. Nonetheless, we have not found any study investigating the impact of the processes on the bioavailability of the bioactive compounds potentially responsible for the functional value of foods.

For the bioactive compounds to be effective in the human organism, they need to be released during digestion and then absorbed in a certain amount in the intestine. Bioavailability is commonly defined as the fraction of a given food constituent that reaches systemic circulation [1]. As to be bioavailable, the compound must be bioaccessible in the first instance, which implies that it needs to be released from the food matrix in the gastrointestinal lumen to become available for intestinal absorption [2]. There are numerous studies related to the in vitro bioaccessibility of different food compounds. However, the in vitro methods possess numerous constraints mainly related to the contribution of the whole human metabolism in the conversion or inactivation of different active forms of the biocompounds [3]. Therefore, in vivo studies are required for the bioavailability knowledge of these compounds. Unfortunately, this information is significantly scarce because of the inherent complexity of clinical trials, especially those involving humans.

The bioavailability of bioactive compounds depends, apart from the mastication and the level of intake, on its nature, the composition and rheological properties of the food matrix and the interactions among the different components, among others [1,3,4,5]. From this point of view, different processes applied for the production of food may have a different impact on some of these aspects. In fact, several studies describe a better bioaccessibility of phenolic compounds after the application of thermal and non-thermal processes [6,7]. In the case of plant foods, in which carbohydrates are the major nutrients, the presence of fibers needs special consideration. Their insoluble fraction is markedly resistant to degradation in the upper gut, which makes the liberation of the active constituents that may be linked to them difficult [1,3]. On the other hand, soluble dietary fiber can also negatively affect the absorption of nutrients because of gel formation, increased viscosity, or binding and entrapment [8,9]. Entrapment of VC in the food matrix, and premature degradation or inhibition by other food components may decrease its bioavailability [10]. It is reported that 5–10% of the total intake of flavonoids, mostly those with monomeric and dimeric structures, can be absorbed in the small intestine, often after deconjugation reactions such as deglycosylation [10]. The absorption of phenolics linked to insoluble dietary fibers becomes ineffective in the small intestine, although these compounds are fermented by gut microbiota in the large intestine, providing other metabolites with different physiological significance; however, a positive interaction between pectin and quercetin has also been described [1,11]. Consequently, positive, null and negative effects of dietary fibers on bioavailability/bioaccessibility of phenolic compounds have been described [1]. In addition to these, the positive effects of phenolic-vitamin interactions on the bioaccessibility/bioavailability of the phenolic compounds and the negative effects on the bioavailability of VC have also been described [1,12,13]. From this point of view, plasma antioxidant capacity has been proposed as a biomarker of food’s antioxidant absorption, providing more valuable biological information than the determination of single antioxidants [14]. The concept of antioxidant capacity describes the ability of redox molecules in foods and biological systems to scavenge free radicals, and it considers the additive and synergistic effects of all the present compounds. Therefore, it may be useful for studying the potential health benefits of antioxidants on oxidative stress-mediated diseases.

The biological functions of VC depend upon its ability to act as an electron donor. Among these, it acts as a cofactor for a variety of enzymes with critical functions throughout the body, functions as a highly effective water-soluble antioxidant, and it is suggested that is involved in the regeneration of vitamin E in vivo [15,16]. Reported beneficial effects of this vitamin include increased baroreflex sensitivity, improved endothelial vascular function, augmented inotropic and thermogenic response to beta-adrenergic stimulation, decreased systemic inflammation and reduced fluid requirements during recovery from thermal injury, improved fatigue resistance, increased iron bioavailability and decreased risk of cardiovascular diseases [17,18,19]. Severe VC deficiency produces scurvy. Despite humans being unable to synthesize their own VC, it must be easily obtained from the diet, principally through adequate fruit and vegetable consumption [15].

Phenolics are secondary metabolites produced by plants against stress conditions such as injury, infection, and UV radiation [20]. Numerous compounds such as phenolic acids, acetophenones, phenylacetic acid, hydroxycinnamic acids, coumarins, naphthoquinones, xanthons, stilbenes, and flavonoids belong to the phenolics compound. Their chemical structure can range from very simple molecules to very complex ones [20]. In recent years, the antioxidant properties of phenolics compounds and their preventive and supportive effects on several diseases, including cancer [20], diabetes, bone, skin, and heart diseases, have been discovered by researchers [9]. It has also been pointed out in the literature that some phenolic compounds promote the growth of useful bacteria and may inhibit the growth of pathogenic bacteria [20]. In this sense, fermentation by lactic acid bacteria can cause changes in both the content and profile of different bioactive compounds that can lead to a change in their bioaccessibility and bioavailability [21].

Grapefruit is a source of VC and phenolics, flavonoids among them. Specifically, the main flavonoids are glycosides naringin, followed by narirutin [22]. As reported by Manach [23], glycosides are not absorbed as such but are hydrolyzed into aglycones which are recovered in plasma. In the case of grapefruit, naringin and narirutin will be converted to aglycone NAG, which is really scarce in this fruit. So naringenin, if absorbed after the ingestion of grapefruit, would be the major flavonoid recovered in plasma. As far as the grapefruit juices considered in this study are concerned, it is convenient at this point to highlight some differences in the processes applied to obtain the corresponding powdered products, which, to some extent, affect their composition [24]. Uscanga et al. [24] made a comparison between FD products obtained from orange puree and from orange squeezed. The authors conclude that the former contains more natural fiber from the fruit, which provides a greater amount of insoluble solutes that contribute to obtaining a less porous powder which is more viscous after rehydration. In addition, in this study, a contribution of the insoluble part of this fiber to the retention of the carotenoids was observed. In this regard, it has also been reported that 2.5% of the insoluble dietary fiber is composed of polyphenols, mainly condensed tannins and flavonoids [25,26]. Furthermore, the very different particle size of the powders obtained by FD and SD affects the viscosity of the juices [27] and both could affect the bioaccessibility of the bioactive compounds [28].

This study was designed given the interest in providing more and more information on the human bioavailability of different bioactive compounds and the scarce information available on this subject. The objective was to provide information on the bioavailability of VC and the main flavonoids in grapefruit juice, recovered as NAG in circulating blood, depending on the process used to obtain it.

## 2. Results

### 2.1. Blood Serum Vitamin C Level

**Figure 1:**
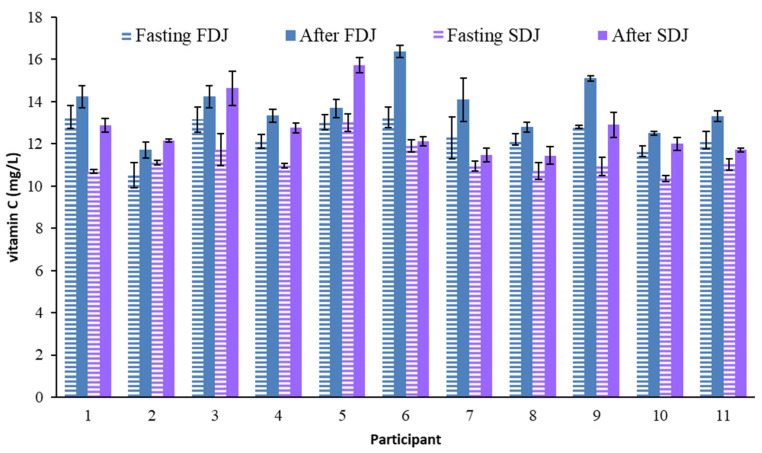
Mean value and standard deviation of serum blood VC concentration analyzed in the participants on the days of consuming reconstituted freeze-dried (FDJ) and spray-dried (SDJ) juices, both fasting and 4 h after the intake of each juice.

Figure 1 shows the VC concentration found in blood serum. Taking into account all the data obtained for each participant, this compound showed a maximum coefficient of variation (CV) of 8%, below the 15% accepted for biological samples following AOAC criteria [29]. The minimum CV being 0.6%. The fasting serum VC values of the participants in the trial varied between 10.1 and 13.6 mg/L. As expected from the country of habitual residence of the participants, all of them were above the adequate level of plasma VC (i.e., 50 µmol/L [13] = 8.80 mg/L), with no hypovitaminosis case detected.

**Figure 2:**
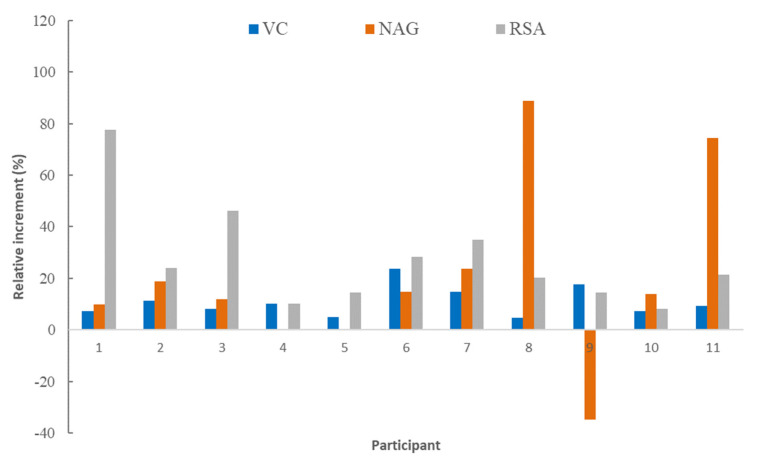
Relative increase, related to fasting level, in serum blood vitamin C (VC), naringenin (NAG), and radical scavenging ability (RSA) due to reconstituted freeze-dried juice intake by each participant.

**Figure 3:**
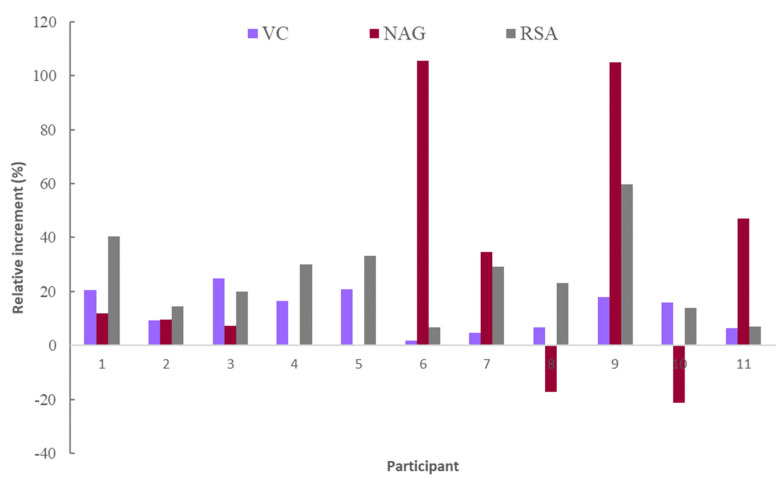
Relative increase, related to fasting level, in serum blood vitamin C (VC), naringenin (NAG), and radical scavenging ability (RSA) due to reconstituted spray-dried juice intake by each participant.

The level of VC in the blood serum of all the participants in the trial increased after the ingestion of each juice, the level reached varying between 11.1 and 16.6 mg/L (Figure 1). This increase, compared to the basal fasting level, evidenced the absorption of VC from juices. When comparing the circulating concentration of VC fasting with that of 4 h after ingestion of the juices, an increase between 0.17 and 3.27 mg/L was observed. The ratio of this increase to the fasting level for each participant is shown in Figure 2 and Figure 3 for FDJ and SDJ, respectively, the relative increases being in the range 4.7–23.6% (FDJ) and 1.8–24.9% (SDJ).

In order to compare the blood VC increase caused in each participant by grapefruit juice ingestion, a multifactor analysis of variance (ANOVA) was carried out with the factors juice and participant. No significant differences (*p* > 0.05) were observed in any case. In this way, the mean 10.85% relative increase of blood circulating VC in the participants in the trial caused by FDJ ingestion was not significantly different (*p* > 0.05) to the mean 13.23% detected after SDJ ingestion (standard error 2.5). As far as the participants are concerned, the mean relative increase caused by the two juices tested was not significantly different (*p* > 0.05) in any of them, the lowest relative increase being 5.7% and the highest 17.9% (standard error = 0.06). In this way, a relative increase of VC in the blood due to juice intake of 12% can be assumed as a grand mean of the 22 average values considered.

### 2.2. Blood Serum NAG Level

**Figure 4:**
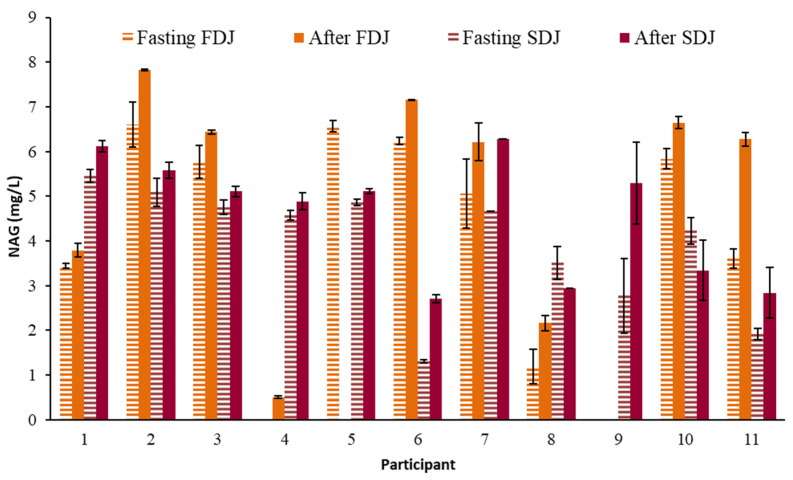
Mean value and standard deviation of serum blood naringenin (NAG) concentration analyzed in the participants on the days of consuming reconstituted freeze-dried (FDJ) and spray-dried (SDJ) juices, both fasting and 4 h after the intake of each juice.

The concentration of NAG analyzed in the serum of the different participants before and after the intake of each juice is shown in Figure 4. In this case, seven of the NAG data showed CV > 15%, a greater number of cases than for VC. Mean values of NAG fasting, after intake of FDJ or SDJ were 4.2 ± 0.8, 5.2 ± 0.4 and 4.6 ± 0.9 mg/L, these values being slightly higher than the 1.6 mg/L than those reported by Manach [20] for NAG absorption from grapefruit juice. The relative increase of NAG after ingestion of the juices (Figure 2) varied between −35 and 106%, being a much wider range of variation than in the case of the VC. The multifactor ANOVA carried out indicated, in all cases, the absence of significant differences (*p* > 0.05) per juice and per participant. In this way, the intake of the juices supposed a mean relative increase of NAG in circulating blood related to fasting value of 28%, although with high variability.

### 2.3. Serum RSA

**Figure 5:**
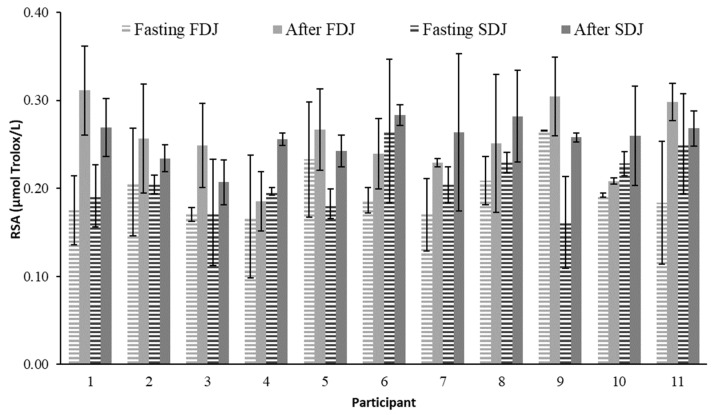
Mean value and standard deviation of serum blood radical scavenging ability (RSA) analyzed in the participants on the days of consuming reconstituted freeze-dried (FDJ) and spray-dried (SDJ) juices, both fasting and 4 h after the intake of each juice.

Values obtained for RSA in the blood serum are shown in Figure 5. Participants in the trial had RSA fasting serum values ranging from 0.16 ± 0.05 to 0.266 ± 0.003 µM Trolox/L. After consuming each juice, the RSA in each participant’s blood serum increased, reaching a level ranging from 0.21 ± 0.02 to 0.31 ± 0.05 µM Trolox/L (Figure 5). This rise compared to the basal fasting level demonstrated the absorption of bioactive substances with antioxidant capacity from juices. An increase in RSA was observed between 0.016 and 0.136 µM Trolox/L after ingestion of the juices. According to Figure 2 and Figure 3, the relative increase of RSA related to fasting level was in the range 6.7–77.7% (CV < 9%). There were no significant differences between the relative increases in RSA obtained either per participant or following FDJ or SDJ consumption (*p* > 0.05). Therefore, the mean relative increase of RSA blood serum caused by FDJ consumption in the participants in the research (27%, standard error 5) did not differ significantly (*p* > 0.05) from the mean generated by SDJ consumption (25%, standard error 5). Thus, the intake of juices resulted in an average relative increase of 26% in RSA compared to the fasting value.

To explain the relationships in the relative increases of the bioactive compounds quantified in blood serum with the RSA, Pearson correlation analyses were performed. Only VC showed a positive significant correlation with RSA (r = 0.5099, *p* < 0.1), suggesting that VC ingested may be the main bioactive compound with potential reducing ability in blood serum. In the case of grapefruit and citric products, different authors have also observed that VC played an important role in antioxidant capacity, while flavonoids may not be the main bioactive compounds with potential reducing ability [30,31,32,33].

## 3. Discussion

Although all the results obtained in this study indicate, qualitatively, the availability of the different compounds studied to perform their function in the organism, it has been attempted to quantify the bioavailability in terms of the amount of compound absorbed per 100 g of compound ingested (Equation (2)). As far as flavonoids are concerned, this bioavailability cannot be calculated for NAG as this compound is not present in the juices ingested by the patients. On the other hand, the VC results analyzed in blood serum showed the least variability and were the only ones that correlated positively and significantly with RSA. Therefore, only the bioavailability of VC was calculated, which in turn could be favored by the presence of phenolic compounds [34].

Grapefruit juices obtained from FD and SD grapefruit, provided to trial participants, contained 34 ± 2 mg VC/100 g and 18.8 ± 0.9 mg VC/100 g, respectively. The different processing the grapefruit underwent to obtain the powder resulted in better retention of VC in the case of FDJ, as has been observed in previous studies [35]. In this regard, considering the amount of juice ingested, participants consumed 136 and 75.2 mg VC, depending on whether the juice was prepared from FD or SD powder, respectively. However, as bioavailability refers to each gram of VC ingested, the results of this parameter allow us to compare whether this compound is better absorbed depending on the processing of the fruit.

**Figure 6:**
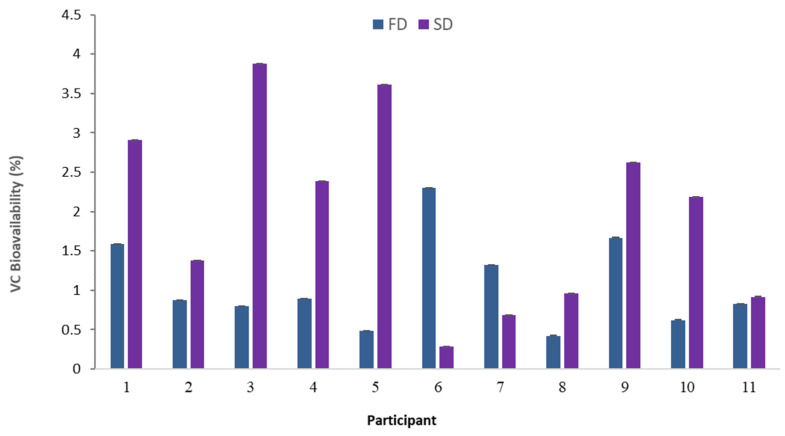
Vitamin C (VC) bioavailability (%) when ingested reconstituted freeze-dried (FDJ) and spray-dried (SDJ) juices. Values referred to mg VC recovered in the blood serum by 100 g of VC ingested.

Figure 6 shows the calculated bioavailability values. These ranged from 0.3 to 3.9 mg VC absorbed/L serum per 100 mg VC ingested, both extremes corresponding to SDJ. The mean values were 1.07 ± 0.15% for FDJ and 1.98 ± 0.03% for SDJ. This range includes the values obtained by other authors who point to 1.76 mg/L serum per 100 mg of VC ingested [36]. In this sense, the differences in the insoluble fiber content and the viscosity of both juices mentioned in the introduction, higher in FDJ, seem to decrease the bioavailability of VC. However, the differences were not large enough to be statistically significant (*p* > 0.05) considering both participants and processes.

## 4. Materials and Methods

### 4.1. Grapefruit Juices

Rehydration of the grapefruit powder obtained by freeze-drying (FDJ) and by spray-drying (SDJ) was carried out to prepare the two juices considered in this study. The same grapefruit (*Citrus paradise* var. Star Ruby) batch, purchased in February 2016 from ANECOOP, a second-tier cooperative located in Valencia (Spain), was used to obtain both powdered products. Grounded and liquidized grapefruit pulp was used for FD and SD, respectively. In both cases, a mix with gum Arabic (GA) and bamboo fiber (BF) was used as to improve the powder characteristics in terms of getting the lowest water content, hygroscopicity, luminosity, color change and the highest content of total phenolic, total carotenoids, VC and RSA, despite the highest powder yield [35]. Briefly, peeled and grounded grapefruit was mixed with 4.2 g GA + 0.6 g BF/100 g grapefruit pulp to obtain FDJ or with 4.3 g GA + 2.1 g BF/100 g liquidized grapefruit to obtain SDJ. No shelf temperature was applied during FD, and the inlet air temperature for SD was 120 °C. FD cake was crushed (Thermomix^®^, Vorwerk, Spain) to obtain a powder which was, in the same way as spray-dried powder, vacuum packed and stored under refrigeration until used.

Each powder was rehydrated on the day of the trial, two hours prior to providing them to the participants. The water added to the powders was calculated as to ensure juices offered with the same amount of grapefruit own’s solutes present in the grapefruit batch used for the study. The corresponding mass balance was applied to this end, taking into account the water mass fraction of the grapefruit (0.867 ± 0.002 g water/g pulp), the amount of GA and BF added to the prepared mix and the water mass fraction of the obtained powders (0.0304 ± 0.007 g water/g FD powder and 0.0186 ± 0.0010 g water/g SD powder).

### 4.2. Selection of Participants in the Study

Ethical approval for the trial was obtained from the Ethics Committee of the Universitat Politècnica de València (Valencia, Spain). A total of 11 healthy volunteers from different countries in South America and Europe, who met the inclusion and exclusion criteria established for the study, were recruited for the trial. Inclusion criteria included age within the range of 18–40 years, with a normal body mass index (BMI between 19 and 29 kg/m^2^). Exclusion criteria included smokers and people with special dietary habits (vegetarians, vegans, macrobiotics, etc.), allergic to any food, pregnant or with the intention to be in the trial period, medicated (includes dietary supplements: vitamin, protein, etc.), suffering infectious diseases by blood (positive serology for hepatitis B virus and C or human immunodeficiency virus), presenting some intestinal malabsorption syndrome, suffering metabolic diseases (diabetes, dyslipidemia, thyroid disorders, etc.) and suffering cardiovascular or kidney diseases (high blood pressure, renal insufficiency, etc.). All the 11 selected participants signed the corresponding informed consent after being explained about the nature, purpose and risks of the study.

### 4.3. Experimental Design and Blood Sample Collection

A crossover design was considered for the clinical trial, so that each subject serves as his/her own matched control, thus blocking the variability due to the subjects. This design allows for a smaller number of participants in the study than in a parallel one while ensuring the same statistical power. All 11 participants were cited on two different days, one for FDJ consumption and another one for SDJ, in different time periods separated by 15 days. Each day, fasting blood samples were obtained from each participant. They were then given 400 g of the juice to be ingested with no meal. After 4 h, during which they were not allowed to eat anything, a second blood draw was performed. Two blood samples from each participant were collected into BDVacutainer SSTII Advance Tubes (REF 367953), which were allowed to coagulate for 30 min and after centrifuged at 20 °C and 1500× *g* for 10 min to separate the serum. The serum concentration of VC, NAG, and RSA was analyzed in duplicate in each tube, as described below. The mean value (and standard deviation) of the two replicates of each of the two tubes from each patient was considered as the corresponding result.

### 4.4. Vitamin C Analysis

To quantify the VC (ascorbic plus dehydroascorbic acids) present in serum blood, 0.5 mL serum was mixed with 1 mL of a 20 g/L DL-dithiothreitol solution for 2 h at room temperature and in darkness. Afterwards, 1 mL of this mixture was extracted with 2.25 mL 0.1% oxalic acid for 3 min and immediately filtered through a 0.45 mm membrane filter before injection. The HPLC (Jasco, Madrid, Spain) conditions were: Ultrabase-C18, 5 mm (4.6 × 250 mm) column (Análisis Vínicos, Tomelloso, Spain); mobile phase 0.1% oxalic acid, volume injection 20 µL, flow rate 1 mL/min, detection at 243 nm and at 25 °C. A standard solution (Panreac, Barcelona, Spain) was prepared. The results were expressed as mg of VC per L of serum. The VC content of the juices served to the participants was also analyzed as described by Galindo et al. [37].

### 4.5. Blood Serum Naringenin Analysis

Grapefruit NAG aglycone was selected to be quantified in serum blood samples. The extraction of flavonoids in biological samples was carried out following the procedure proposed by Manach et al. [23] with some modifications. Next, 0.5 mL of serum were acidified with 50 µL of acetic acid to pH 4.9 and incubated for 18 h at 37 °C in the presence of β-glucuronidasa hydrolyzed with sulphatase. Samples were mixed with 4 vols of MeOH and centrifuged 10 min at 9400× *g* (SelectaMedifriger-BL, 4 °C) to obtain the supernatant that was filtered through a 0.45 µm membrane filter. The HPLC method and instrumentation was: Ultrabase C18, 5 µm (4.6 × 250 mm) column (Análisis Vínicos, Tomelloso, Spain); mobile phase was composed of methanol and water, and linear gradient elution was performed starting at 30:70 (*v*/*v*) to reach 100:0 (*v*/*v*) methanol: water at 70 min, volume injection 25 µL and flowrate 1 mL/min. Chromatograms were recorded at 286, 284 and 254 nm and at 25 °C. The standard curves of the reference NAG (Extrasynthese, Genay, France) were used to quantify the flavonoids. Naphthalene was used as an internal standard [22]. The results were expressed as mg of each flavonoid per L of serum.

### 4.6. Radical Scavenging Activity

The most widely used methods for measuring antioxidant capacity involve the generation of radical species, the presence of antioxidants determining the disappearance of these radicals. DPPH (2,2-diphenyl-1-picrylhydrazyl) radical assay examines the availability of a substance to reduce the DPPH˙ radical. As DPPH˙ is one of the few stable and commercially available organic nitrogen radicals, it is preferred over other methods in which the radical has to be formed [38]. In this study, RSA was determined spectrophotometrically by the modified procedure proposed by Martínez et al. [39], using the DPPH method. Briefly, 0.2 mL of serum was deproteinated by adding of 0.7 mL of methanol, vortexing for 30 s and centrifuging at 9400× *g* for 30 min (Eppendorf centrifuge 5804R, Germany) to separate the proteins. Next, 0.3 mL of the supernatant was added to 1.2 mL of 0.076 mmol/L DPPH solution (diluted in MeOH, Sigma-Aldrich, Taufkirchen, Germany) and mixed thoroughly. Absorbance was read when the reaction was stabilized, after 5 min, at 517 nm (Thermo Electron Corporation, Waltham, MA, USA) against the blank prepared in an identical way, without the addition of the sample. The percentage of DPPH was calculated according to Equation (1). The final results were expressed as mmol Trolox equivalent (TE), using a Trolox calibration curve (Sigma-Aldrich, Taufkirchen, Germany).

(1) $$\%DPPH=\frac{A_{blank}-A_{sample}}{A_{blank}}\times 100$$

where: *A_blank_* is the absorbance of the blank and *A_sample_* is the absorbance of the sample.

### 4.7. Vitamin C Bioavailability

For VC, bioavailability was quantified in terms of the amount of the compound absorbed per 100 g of the compound ingested ($i_{VC}$), based on serum VC concentrations before ($C_{VC}^{b}$) and after ($C_{VC}^{a}$) ingestion of the juice (Equation (2)).

(2) $$VC\;Bioavailability\;(\%)=\frac{C_{VC}^{a}-C_{VC}^{b}}{i_{VC}}\times 100$$

### 4.8. Statistical Analysis

For VC and NAG, the serum concentration before and after the juices’ intake, also as the corresponding relative change, were considered as the response variables, together with the corresponding values of RSA. To assess the differences between samples, ANOVA was performed for these response variables taking into account the factors participant and juice, with a total of 11 participants and two juices tested. Fisher’s least significant difference (LSD) procedure with a 95% confidence level (*p* < 0.05) was used to this end. The same ANOVA was carried out for VC bioavailability. Statgraphics Centurion XVI.II for Windows was used for the statistical analysis.

## 5. Conclusions

VC and the main flavonoids present in juices prepared by rehydration of grapefruit powder obtained by FD and SD are absorbed by the human body, which supposes an increase in the RSA of the blood serum. In no case, significant differences (*p* > 0.05) due to either the test participants or the processes used to obtain the powder were observed. Thus, it can be concluded that the intake of grapefruit juices resulted, on average, in a relative increase in VC, NAG and RSA of 12%, 28% and 26%, respectively. VC showed a positive and significant correlation with RSA, which was not detected with NAG. It was possible to quantify the bioavailability of VC, which on average was found to be 1.529 ± 0.002 mg VC/L serum per 100 mg of VC ingested.

## Data Availability

Not applicable.
